# Supplementary material for: Analysis of PPARγ Signaling Activity in Psoriasis
Source: Int J Mol Sci. 2021 Aug 10;22(16):8603. doi: 10.3390/ijms22168603 (PMC8395241; doi:10.3390/ijms22168603)
Supplement: Supplementary file 1 [file ijms-22-08603-s001.zip › Supplemental materials_Analysis of PPARg signaling activity in psoriasis/Pathway models/Models images and html files/PPARG expressed in cells assotiated with psoriasis/225693.html]

B-cell


# CellType B-cell

|  |  |
| --- | --- |
| URN | urn:agi-ncimcelltype:C0004561 |
| Total Entities | 78 |
| Connectivity | 10498 |
| Name | B-cell |

---

|  |  |
| --- | --- |
| ChildConcepts | B cell function |
|  | B-cell homeostasis |
|  | B cell apoptosis |
|  | B-cell differentiation |
|  | B-cell proliferation |
|  | B-cell chemotaxis |
|  | mature B-cell |
|  | immature B-cell |
|  | transitional B-cell |
|  | B-cell development |
|  | B-cell fate commitment |
|  | B-cell selection |
|  | B-cell survival |
|  | B-cell growth |
|  | B-cell repertoire |
|  | B-cell clonal anergy |
|  | B-cell interaction |
|  | B-cell infiltration |
|  | B-cell adhesion |
|  | B-cell homing |
|  | B-cell migration |
|  | B-cell phenotype |
|  | B-cell population |
|  | B-cell formation |
|  | B-cell priming |
|  | B-cell renewal |
|  | B-cell aggregation |
|  | B-cell viability |
|  | B-cell motility |
|  | B-cell destruction |
|  | B-cell sequestration |
|  | B-cell communication |
|  | B-cell localization |
|  | B-cell cytotoxicity |
|  | B-cell fate |
|  | B-cell division |
|  | B-cell polarity |
|  | B-cell spreading |
|  | B-cell transendothelial migration |
|  | B-cell distribution |
|  | B-cell compartmentalization |
|  | B-cell fate specification |
|  | B-cell death |
|  | B-cell mediated cytotoxicity |
|  | B-cell size |
|  | B-cell senescence |
|  | B-cell swelling |
|  | B-cell fate determination |
|  | B-cell extravasation |
|  | B-cell proliferative response |
|  | B-cell recognition |
|  | B-cell dedifferentiation |
|  | B-cell developmental process |
|  | B-cell contact |
|  | B-cell motion |
|  | B-cell transdifferentiation |
|  | B-cell rolling |
|  | B-cell damage |
|  | B-cell lifespan |
|  | B-cell regeneration |
|  | B-cell macrophage differentiation |
|  | B-cell autophagy |
|  | B-cell structure |
|  | B-cell agglutination |
|  | B-cell disruption |
|  | B-cell tethering |
|  | B-cell elongation |
|  | B-cell invasion |
|  | B-cell ratio |
|  | B-cell T-cell homeostasis |
|  | B-cell diapedesis |
|  | B-cell stress |
|  | B-cell volume |
|  | B-cell ultrastructure |
|  | B-cell phagocytosis |
|  | B-cell morphogenesis |
|  | B-cell architecture |
|  | B-cell enlargement |

---

|  |  |
| --- | --- |
| Pathway | Hematopoietic Cell Lineage: B-cell (mouse) |
|  | Hematopoietic Cell Lineage: B-cell |
|  | Tumor Infiltrating Macrophages in Cancer Progression and Immune Escape |
|  | Proteins/Ions Transporters -> Expression Targets in Lymphoid System and Blood |
|  | LDLR -> Expression Targets in Lymphoid System and Blood |
|  | TGF-beta Receptors Family -> Expression Targets in Lymphoid System and Blood |
|  | EGFR-> Expression Targets in Lymphoid System and Blood |
|  | Elevated Receptors -> Expression Targets in Lymph Node |
|  | Elevated Receptors -> Expression Targets in Lymphoid System and Blood |
|  | F2R/F2RL1 -> Expression Targets in Lymphoid System and Blood |
|  | CD Markers -> Expression Targets in Lymphoid System and Blood |
|  | VIPR1/VIPR2 -> Expression Targets in Lymphoid System and Blood |
|  | GPCRs Family -> Expression Targets in Lymphoid System and Blood |
|  | TNF Receptors -> Expression Targets in Lymphoid System and Blood |
|  | Immunoglobulin Superfamily -> Expression Targets in Lymphoid System and Blood |
|  | Receptor Tyrosine Kinase Family -> Expression Targets in Lymphoid System and Blood |
|  | IgE Receptors -> Targets in Lymphoid System and Blood |
|  | Chemokine Receptor Family -> Expression Targets in Lymphoid System |
|  | PPARG expressed in cells assotiated with psoriasis |
|  | Neighbors of B-cell |
|  | Overview. Diseases of the skin (chapter 11) |
|  | 1\_Th2 cell response in asthma |
|  | 1\_CD4+ T cell and epithelial cell response in celiac disease |
|  | 3\_Islet beta-cell destruction in T1D |

---

|  |  |
| --- | --- |
| MedScan ID | 10000186 |

---

|  |  |
| --- | --- |
| Alias | B-cell |
|  | bone marrow lymphocyte |
|  | B-lymphocytic |
|  | bursa derived lymphocytes |
|  | B-lymphocyte subsets |
|  | bone marrow derived lymphocyte |
|  | bone marrow lymphocytes |
|  | B-lymphocytes |
|  | bursa derived lymphocyte |
|  | B-cells |
|  | bone marrow derived lymphocytes |
|  | B-lymphocyte |
|  | bursa-dependent lymphocytes |
|  | bursa-dependent lymphocyte |

---

|  |  |
| --- | --- |
| NCIm ID | C0004561 |

---
